# In Vitro Culture of Mammalian Embryos: Is There Room for Improvement?

**DOI:** 10.3390/cells13120996

**Published:** 2024-06-07

**Authors:** Roberto Gualtieri, Vincenza De Gregorio, Andrea Candela, Angela Travaglione, Vincenzo Genovese, Vincenza Barbato, Riccardo Talevi

**Affiliations:** Department of Biology, University of Naples ‘’Federico II’’, Complesso Universitario Di Monte S. Angelo, Via Cinthia, 80126 Naples, Italy; vincenza.degregorio@unina.it (V.D.G.); andrea.candela@unina.it (A.C.); angela.travaglione@unina.it (A.T.); vincenzo.genovese@unina.it (V.G.); barbato_vincenza@libero.it (V.B.); riccardo.talevi@unina.it (R.T.)

**Keywords:** preimplantation embryo development, maternal reproductive tract, in vitro embryo culture, developmental competence, dynamic culture

## Abstract

Preimplantation embryo culture, pivotal in assisted reproductive technology (ART), has lagged in innovation compared to embryo selection advancements. This review examines the persisting gap between in vivo and in vitro embryo development, emphasizing the need for improved culture conditions. While in humans this gap is hardly estimated, animal models, particularly bovines, reveal clear disparities in developmental competence, cryotolerance, pregnancy and live birth rates between in vitro-produced (IVP) and in vivo-derived (IVD) embryos. Molecular analyses unveil distinct differences in morphology, metabolism, and genomic stability, underscoring the need for refining culture conditions for better ART outcomes. To this end, a deeper comprehension of oviduct physiology and embryo transport is crucial for grasping embryo–maternal interactions’ mechanisms. Research on autocrine and paracrine factors, and extracellular vesicles in embryo–maternal tract interactions, elucidates vital communication networks for successful implantation and pregnancy. In vitro, confinement, and embryo density are key factors to boost embryo development. Advanced dynamic culture systems mimicking fluid mechanical stimulation in the oviduct, through vibration, tilting, and microfluidic methods, and the use of innovative softer substrates, hold promise for optimizing in vitro embryo development.

## 1. Introduction

Although several studies contributed to the design of universal or sequential culture media tailored for preimplantation mammalian embryos [1], in vitro embryo culture was essentially unchanged since the beginning of human in vitro fertilization and is still performed in relatively large media volumes under static conditions [2]. It has been shown that numerous factors and mishandlings during standard culture procedures in these systems can pose a threat to the successful development of embryos [3,4]. However, the fundamental culture conditions persist unchanged, as if the inherent biological constraints on blastocyst development from the produced zygotes had already been reached. In comparison to the few efforts devoted to the improvement of the physico-chemical embryo culture environment, a range of technologies and non-invasive markers have been increasingly developed for selecting embryos endowed with the maximal potential to implant and generate live births [5,6,7,8].

Many researchers acknowledge the significant disparities between the physico-chemical conditions of in vitro preimplantation embryo development and the dynamic environments encountered in vivo as the embryo progresses from fertilization in the Fallopian tube to implantation in the uterus. Despite this recognition, there remains uncertainty regarding the extent to which the developmental potential of in vitro cultured embryos is compromised compared to their in vivo counterparts, particularly in humans with limited data compared to animal models. The primary objective of this paper is to review studies focusing on (1) the characteristics and performance of embryos developed in vivo vs. in vitro; (2) the dynamic physico-chemical conditions experienced by embryos during transit and development within the maternal tract; (3) the standard in vitro culture conditions; and (4) the dynamic culture systems designed in research studies and their effects on embryo development.

## 2. Natural versus In Vitro Conceptions in the Human

The efficacy of conception in vivo in humans is a highly debated and speculative topic based on relatively few old data. We still do not have definitive estimates of embryo mortality from fertilization to birth during in vivo conception in a fertile women cohort. Moreover, since the earlier marker of pregnancy is represented by a rise in hCG, we have different figures of what occurs from the second week of gestation, i.e., from implantation to birth, but still lack evidence on what has been called the black box of early pregnancy loss that covers the period from fertilization to implantation. Speculative estimates of this currently unmeasurable loss in vivo range from 10 to 70% [9,10,11,12,13].

The best estimates of the efficacy of in vivo conceptions in terms of live births per implantation are provided by three old, well-designed studies showing that about two-thirds of the in vivo conceptions detected by elevated hCG at least 1 week after ovulation result in a live birth [14,15,16]. Since these studies do not measure the pre-implantation embryo loss, embryo mortality from fertilization to birth in the human is still not known. Although speculative estimates of embryo loss before and during implantation range from 30 to 75% [17], it is currently accepted that up to 30% of conceptions do not complete implantation [18]. Therefore, it has been suggested that about 30% of the in vivo conceptions result in a live birth due to a 30% loss before implantation, a further unrecognized 30% loss after implantation but before the missed menstrual period, and a final 10% loss due to clinical miscarriage [18]. The highest estimates of live births during natural conception have been recently provided by Jarvis et al. (2016, 2017) [13,17], who re-analyzed the aggregate data from the three reliable hCG studies. Jarvis hypothesized that the criteria adopted to select fertile women cohorts did not ensure the exclusion of a proportion of sub-fertile or infertile couples. Therefore, he identified sub-cohorts of sub-fertile women to estimate the decline in pregnancy rates across successive cycles. By excluding these sub-cohorts from the initial population of women, it was determined that 40–60% of conceptions resulted in live births when considering only presumably fertile women. While human reproduction in vivo appears relatively inefficient compared to other mammals [19], studies measuring reproductive efficiency on an egg-by-egg basis suggest even lower success in vitro [20,21,22]. However, comparing reproductive efficiency in vitro vs. in vivo requires consideration of several biases. In vivo estimates typically involve unstimulated, fertile women within the appropriate reproductive age range. Conversely, patients undergoing assisted reproduction are sub-fertile and more reproductively aged. According to Patrizio and colleagues (2007) [22], only about 5% of harvested mature oocytes led to live births. However, this analysis was based on 31 cycles involving 26 patients (with a mean age of 37 ± 3.7 years), including 10 cycles with recurrent pregnancy loss, 15 cycles with advanced maternal age, and 6 cycles for unexplained recurrent pregnancy failure. Jones et al. (2010) [23] reported a 6% efficiency of human reproduction in vitro for an age group of 25–29 years and suggested that IVF is only one-fifth as efficient as natural human reproduction. In conclusion, the differences between in vivo and in vitro conceptions in humans are still inconclusive due to the scarcity of data available in literature.

## 3. Natural versus In Vitro Conceptions in Animal Models

The success of the basic and straightforward embryo culture conditions in assisted reproductive technology (ART) is demonstrated by the birth of over 10 million babies. However, a fundamental yet unanswered question persists: to what extent do current in vitro embryo culture conditions replicate those experienced by embryos during in vivo development? In other words, is there room for improvement in the developmental rates and quality of in vitro-produced embryos to enhance clinical success rates?

While data from human ART are inconclusive, more stringent and unbiased experiments have been conducted using animal models. Studies in the mouse model showed several differences between in vitro-produced (IVP) and in vivo-derived (IVD) embryos. IVP embryos had altered ICM and TE cell numbers, lower hatching rates, increased apoptosis, lower cryotolerance, altered metabolism and oxidative stress, and differential gene expression (Table 1) [24,25,26,27,28,29,30].

Bovine is considered a more suitable animal model for humans in many respects.

In vitro embryo culture conditions in both bovines and humans exhibit striking similarities, with many of the technologies initially developed in cattle serving as the foundation for human ART [24]. Furthermore, bovine and human embryo development display more similarities than those observed between human and murine embryonic development [25,26,27,28]. Bovines are excellent models for studying early embryo–maternal communication in humans due to their analogous cycle length, pregnancy duration, usual single offspring, and genetic imprinting patterns [29,30]. Bovine reproduction has been extensively studied for its commercial significance and the lack of ethical constraints that limit experimental studies on human embryos. A unique opportunity for comparing the competence of IVP and IVD bovine embryos arises from data collected in the cattle embryo industry. The 31st annual report of the International Embryo Technology Society (IETS) revealed that over 2 million embryos were collected or produced in cattle in 2021 [31]. Although the number of transferrable IVP embryos exceeded that of IVD embryos for the first time in 2016 [32], accounting for the 79.7% of all transferrable cattle embryos in 2021 [31], the pregnancy and live birth rates of IVP embryos remain lower compared to their in vivo counterparts. On average, pregnancy rates after transfer of IVP or IVD bovine embryos are 40.1% and 64.1%, respectively [33], demonstrating that the competence of IVP embryos consistently lags behind that of IVD embryos [34]. In addition, IVP embryos have a reduced cryotolerance and a higher lipid accumulation compared to IVD embryos [35]. This is also exemplified by the IETS 2021 data showing that frozen IVD embryo transfer is still preferred compared to frozen IVP embryo transfer (60.5% vs. 41.3%) [31]. During development, lipids are used by the mitochondria to increase the ATP production and promote compaction and blastocyst formation [36]. However, lipids can accumulate in the embryo due to an increased uptake from the culture environment [37] or to a decreased activity of mitochondria [38]. Gad et al. (2012) [39] reported that embryos cultured in vitro at the time of embryonic genome activation are particularly affected by a down-regulation of lipid metabolism-related genes compared to embryos developed in vivo. In agreement with those findings, bovine embryos generated by IVM/IVF followed by in vivo culture in the ewe oviduct had similar cryotolerance and pregnancy rates after frozen embryo transfer than IVD embryos [40]. Havlicek et al. (2009) [41] using zygotes produced in vitro and then cultured in vivo also demonstrated that the duration of in vivo culture is crucial for cryotolerance. On the cellular and molecular level, IVP bovine embryos differ from their in vivo counterparts in many respects. Clear differences in morphology and ultrastructure [42,43,44,45], inner cell mass/trophectoderm allocation [46], intercellular connectivity [47], lipid content and profiles [48], energy metabolism [49,50,51], transcriptomic [39,52,53,54], proteomics [55], chromosome aberrations [56,57], and methylation patterns [58] underlie the higher developmental competence of IVD compared to IVP embryos. Rizos et al. (2002) [45] demonstrated that in vitro embryo culture and in vivo development produced similar blastocyst rates, but IVD blastocysts had a dramatically higher competence as shown by the increased survival after cryopreservation. Differences between IVP and IVD bovine embryos are summarized in Table 2.

The contribution of the natural environment to the embryo competence is also exemplified by the enhanced quality of IVP embryos when cultured short- or long-term in vivo in homologous or heterologous oviducts [40,41].

In conclusion, data in animal models highlight clear differences between the consequences of in vivo and in vitro environment on embryo developmental competence.

## 4. Can In Vitro Culture Increase Embryo Aneuploidy?

### 4.1. Human

Embryo aneuploidy is a major contributor to low human fecundability both in vivo and in vitro. The overall incidence of aneuploidy in newborns following natural conception is less than 1% due to the progressive elimination of aneuploid embryos through pre- and post-implantation loss. Nondisjunction of bivalents and premature separation of sister chromatids during oocyte’s meiosis is responsible for embryo homogeneous aneuploidies which are maternal age-dependent both in vivo and in vitro [59,60]. However, errors during the post-zygotic mitotic divisions give rise to an impressively high incidence of age-independent mosaic embryo aneuploidies [61]. Single blastomere analysis of ART-generated embryos demonstrated that more than 75% of them can be mosaics [62,63]. Although it is still a matter of debate, the aneuploidy levels in the general vs. the ART population are hypothesized to be similar [61]. The observation of average euploidy rates ranging from 39.5 to 82.5 on a population of young oocyte donors in 42 different centers raised concerns on the possibility that controlled ovarian stimulation [64] and/or embryo culture systems can affect the euploidy of ART-generated blastocysts [65]. However, several studies did not find different aneuploidy rates in embryos of unstimulated vs. stimulated patients or in embryos from patients subjected to different doses and length of gonadotropins treatment [65,66,67,68]. The possibility that the culture system affects the embryo aneuploidy rate may indicate that the suboptimal microenvironments experienced by the embryo in vitro might induce perturbations in chromosome segregation during the post-zygotic mitosis. Several studies examined a possible correlation between culture settings and embryo aneuploidy. Culture of sibling oocytes under different extracellular pHs (6 vs. 7.5% CO_2_ [69]; 6 vs. 7% CO_2_ [70]) had no impact on blastocyst development but a significantly lower euploidy rate was found at 7 and 7.5% CO_2_. Different single-step embryo culture media reportedly did not affect the embryo euploidy of sibling oocytes [71]. The possible impact of embryo culture systems on euploidy rates in human ART has been recently reviewed by Swain et al. (2021) [72]. Although some data suggest that the culture system may influence the incidence of post-zygotic mitotic errors, we still do not have conclusive indications in the human.

Despite the fact that the euploidy of embryos is a fundamental prerequisite for successful implantation, the clinical significance of PGT-A is still a matter of debate. Clinical evidence demonstrates that transfer of mosaic embryos can result in healthy live births though with lower implantation rates and increased miscarriage rates [73,74,75,76,77,78]. Although the clinical outcome of mosaic embryos depends on the type of aneuploidy involved, available data indicate that transfer of embryos with less than 50% aneuploid cells have better outcomes compared to embryos with higher levels of mosaicism [74,75,77,78,79,80]. Recent findings suggest that mosaic embryos can undergo a self-correction during development through apoptosis of aneuploid blastomeres [81]. In fact, a recent multicenter study demonstrated that the incidence of mosaicism in births from transfer of mosaic embryos is like that recorded after transfer of euploid embryos [77]. PGT-A analyzes the DNA of 5–10 biopsied trophectoderm cells and we still do not know to what extent mosaicism in trophoectoderm cells is representative of true inner cell mass or fetal mosaicism [82]. Since the live birth rate of single euploid blastocyst transfer is limited to 50–60% [83], blastocyst’s euploidy is not sufficient to predict the embryo competence to implant and result in a healthy live birth. Additional markers such as blastocyst morphological grading [84] and developmental speed [85] must be considered to predict the success rates of euploid blastocyst transfer.

### 4.2. Animal Models

More rigorous research, relatively unhampered by the ethical limitations and the innumerous confounding variables present in human studies, can be conducted in animal models.

Although performed with older and technically limited procedures, fluorescent in situ hybridization and comparative genomic hybridization studies suggested a higher incidence of whole chromosome aneuploidy in IVP compared to IVD embryos in ovine, equine, and porcine models [86,87,88].

These data were confirmed and extended in recent studies using more robust procedures able to detect the extent and origin of all chromosomal errors. Tsuiko et al. (2017) [57], using the same parents, compared the genome stability of in vivo vs. in vitro conceived bovine embryos through genome-wide single-cell analysis of dissociated blastomeres. Findings showed the presence of at least one chromosomally aberrant blastomere in only 18.8% of the embryos conceived and developed in vivo, compared to 69.2% of IVF embryos and 84.6% of IVM-IVF embryos. Although this study was carried out on a limited number of cleavage-stage embryos, data clearly highlight the involvement of in vitro embryo culture and in vitro maturation on chromosome instability. Similar findings were demonstrated through single nucleotide polymorphism (SNP)-based PGT-A algorithms in the porcine model by Jochems et al. (2023) [89], who observed more errors in in vitro vs. in vivo-conceived blastocysts (79.7% vs. 13.6%). A study in the equine showed that the incidence of micronuclei which are considered indicators of chromosomal aneuploidy was higher in IVP compared to IVD blastocysts [90]. Taken together, animal data indicate a higher genomic instability in embryos produced in vitro compared to their in vivo counterparts corroborating the need for refining current in vitro embryo culture conditions to mimic more closely the in vivo microenvironment and enhance the success and safety of ART.

## 5. Embryo Transport in the Female Reproductive Tract

During its transport in vivo along the oviduct and the uterus, the embryo undergoes several distinctive biophysical and biochemical interactions that could enhance its ability to reach the blastocyst stage and increase its competence to implant and develop to term, resulting in healthy offspring. Assisted reproduction in humans, farm animals, and studies in animal models that bypass the oviduct to achieve pregnancy have shown that embryo–maternal interactions are indeed dispensable. This may have shifted researchers’ focus away from attempting to more closely mimic the natural environment in which the embryo develops in vivo.

**Figure 1:**
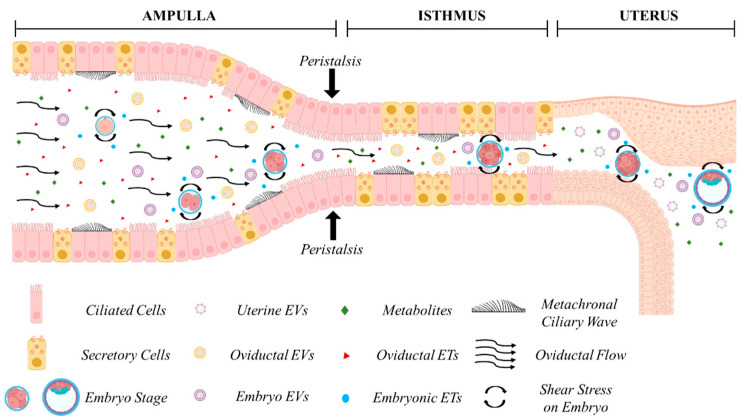
In vivo embryo development in the maternal tract. Cross-talk between the embryo and the maternal tract. Oviductal, endometrial, and embryonic secretions in the form of embryotropins (ETs) and various signaling factors, enveloped or less in extracellular vesicles (EVs), are concentrated in a confined environment. Secretion of maternal fluids, metachronal ciliary waves, and peristaltic contractions exert biomechanical forces on the developing embryo during its journey through the ampulla, isthmus, and the uterus. Figure created with BioRender.com (accessed on 11 April 2024).

The oviduct (Fallopian tube in humans) (Figure 1) is composed by the infundibulum (fimbria in humans), mostly lined by ciliated epithelial cells, that catches the ovulated cumulus–oocyte complex; the ampulla, site of fertilization, where ciliated cells are more represented than secretory cells; the ampullary isthmic junction that separates the lower ampulla by the isthmus; the isthmus, predominantly lined by secretory epithelial cells, and the utero-tubal junction or the intramural segment that divides the isthmus from the uterus. The mucosa is composed of primary, secondary, and tertiary folds in the ampullary segment and only primary folds in the isthmic segment. The underlying connective tissue separates the mucosa by the smooth muscle which is arranged into an inner longitudinal, a middle circular, and an outer longitudinal layer [91]. The smooth muscle is very thin in the ampulla and thick and composed of multiple layers in the isthmus [92].

Understanding the dynamics of embryo transport in the oviduct in vivo is crucial for comprehending the mechanisms underlying oviduct–embryo interactions and optimizing embryo culture in vitro. However, most available data across various species are derived from ex vivo studies, with direct in vivo imaging being rare. The duration of embryo residence in the oviduct varies among species. In humans, egg transport duration has been estimated through the recovery of embryos from the Fallopian tube or uterus following surgical sterilization at known times after the LH surge [92]. Embryos spend approximately 80 h in the oviduct [92], with transport occurring in two phases: a slow phase lasting 72 h in the ampulla, where entry into the isthmus is inhibited by a gate that relaxes under progesterone influence, followed by a rapid phase lasting 8 h in the isthmus [93]. It has been assumed that embryos reach the uterine cavity around the 8–12-cell stage, i.e., at the time of compaction, as 8-cell embryos are the latest stage recovered from the Fallopian tube, and 12-cell embryos are the earliest stage observed in the uterus. Studies in other mammals suggest that embryos develop within the oviduct approximately 4 days after ovulation [94], reaching the uterus around the 16-cell stage in bovine [95], 5.5 days in equine [96], and 4 days in mice [97]. In mice, recent in vivo imaging using optical coherence microscopy (OCM) and optical coherence tomography (OCT) through an intravital window has provided new insights into embryo transport, enabling the determination of developmental stages and in vivo volumetric imaging and tracking of oocytes and preimplantation embryos within the native environment of the mouse oviduct [98,99]. Moore et al. (2019) [98] showed the presence of pronuclear zygotes at 0.5 day post-coitum (dpc) in the ampulla, 2-cell embryos at 1.5 dpc were found in the isthmus, and 4–8-cell embryos at 2.5 dpc were located close to the utero-tubal junction. This is consistent with older studies showing that the mouse embryo enters the uterus approximately 72 h post-coitum (0:00–2:00 h of Day 4), in the morula or early blastocyst stage [97].

The passage of the embryo within the oviduct is believed to be facilitated by three different mechanisms, the production of oviductal fluid, the beating of cilia, and the peristaltic contraction of smooth muscle [10] (Figure 1). The contribution of each factor to embryo descent is still matter of debate [100,101,102], and ciliary beating has typically been regarded as the primary propulsive force involved [101,103,104,105,106]. However, while all factors may exert a biophysical influence on the developing embryo, recent studies have underscored the primary role of muscle contraction in embryo transport. Dixon et al. (2009) [107], using video analyses and Spatio-Temporal Maps, suggested that propagating muscular contractions represent the main propulsive force driving the pendular movement of the egg along the mouse oviduct. They also noted that treatment with an L-type Ca^2+^ channel antagonist suppressed both muscular contractions and egg movement, while ciliary beating remained unaffected. This finding is consistent with the ability of women affected by immotile cilia syndrome to conceive, albeit with reduced efficiency [108,109], and with the fertility of mice deficient in two microRNA clusters lacking cilia but still capable of managing embryo migration from the oviduct into the uterus [110]. Wang and Larina (2020) [99], using in vivo volumetric OCT imaging and tracking of oocytes and embryos in the mouse oviduct, observed unexpected movement patterns, suggesting that egg/embryo transport is more complex than generally assumed. Cumulus–oocyte complexes rotate in groups likely propelled by cilia in the upper ampulla. Groups of fertilized oocytes in the lower ampulla undergo short-distance pulsatile oscillations while descending toward the isthmic-ampullary junction, moving backward when isthmus contracts and forward when it relaxes. At 0.5 days post-coitum (dpc), groups of preimplantation embryos remain stable in the lower ampulla, with average speeds (about 5–70 μm/s) and maximum speeds (20–230 μm/s) depending on the number of embryos present. At 1.5 dpc, embryos in the upper isthmus are relatively spread and individual embryos exhibit very rapid, bi-directional movements (40–740 μm/s) caused by propelling muscular contraction waves. In the lower isthmus, embryos nearing the utero-tubal junctions once again form groups and undergo pulsatile oscillations with reduced speeds (14.7 to 60.0 μm/s). This study highlights that embryos experience different physical forces depending on the timing of transport along the oviduct, which could biomechanically influence embryo development.

## 6. Embryo–Maternal Tract Interactions

During the descent along the female reproductive tract, the preimplantation embryo communicates bidirectionally with oviductal cells of the ampulla, isthmus, and then with endometrial cells. The beneficial effects of culturing embryos with oviductal cells or with their conditioned media were first demonstrated by Gandolfi and Moor (1987) in the sheep [111] and by Eyestone and First (1989) in cattle [112]. Promotion of embryo development through co-culture with autologous or heterologous oviductal cells, uterine fibroblasts, cumulus cells, and cells extraneous to the maternal reproductive tract has been reported in several mammals and the human as well [113,114,115,116,117,118]. These studies prompted the use of co-culture in clinical embryology in human and domestic ruminants to more closely simulate the interaction with the maternal tract in vitro. Co-culture systems have been hypothesized to improve embryo development through (1) the removal of deleterious components of embryo metabolism and reduction in oxidative stress, and (2) active secretions of embryo-trophic factors [119]. However, co-culture has been discontinued for several reasons, potential transmission of pathogens [120], too complex methodology in the clinical context, introduction of reduced oxygen tension which ameliorates blastocyst formation [121], and uncertainty of the beneficial actions.

Along with the presence of maternal factors, the embryo itself secretes embryotropins that promote embryonic development through an autocrine action [122]. Since the oviduct is a virtual cavity, these putative embryotropins should reach a critical concentration required for their positive action on embryo development. The beneficial effects of culturing embryos in groups rather than singly in a defined culture medium volume was recognized more than 30 years ago and has been putatively attributed to the enhanced concentration of embryo-secreted autocrine and paracrine factors [123]. During in vivo conception, putative signaling factors known as embryotropins or embryokines [124,125] can be secreted by the embryo itself or by the epithelial cells lining the female reproductive tracts, but their exact nature remains elusive. Since embryo development in vitro occurs in the absence of maternally-derived signaling factors, embryotropins secreted by the embryo itself and involved in inter-embryonic communication could be responsible for the positive effects of group culture observed in most mammals studied thus far [123,126,127,128,129]. Embryos can communicate with each other or with neighboring embryos through various signaling factors, including proteins, lipids, neurotransmitters, saccharides, nucleotides, and micro RNAs [122]. Studying the proteins released by embryos, however, is complicated by the low concentration of putative embryotropins compared to albumin and other proteins typically added in high concentrations to embryo culture media. Proteins detected in the culture media may stem from lysed cells, which are commonly found not only in arrested embryos but also in developing embryos [130]. Therefore, secretome studies should be conducted on individually cultured high-quality embryos, ensuring the absence of membrane-damaged cells [131]. Within this framework, several studies indicate that preimplantation embryos secrete various factors that may act through autocrine signaling, as they also express the corresponding receptors [122,132].

Extracellular vesicles (EVs) represent an additional recently discovered mechanism through which embryos may communicate with each other and with the female reproductive tract [133]. EVs are nanoparticles surrounded by a lipid bilayer, physiologically released from cells, which contain bioactive molecules such as lipids, proteins, DNA, mRNA, and miRNAs [134,135]. EVs comprise exosomes, microvesicles, and apoptotic bodies, which are characterized by different biogenesis mechanisms and dimensions [135]. EVs participate in cell–cell communication in various biological processes and are relevant modulators during embryo–maternal communication [136].

EVs released by preimplantation embryos, oviductal, and endometrial cells influence the developmental competence and gene expression of embryos, as well as oviductal/endometrial gene expression and functions [137]. Since the oviduct and uterus are considered virtual cavities in which embryos develop in confined fluid volumes, EVs may be present at high concentrations suitable for acting as bidirectional players in embryo–maternal tract cell–cell communication to regulate developmental competence and implantation in vivo. EVs recovered from the human Fallopian tube have been demonstrated to carry miRNAs that increase murine embryo viability in vitro [138]. Moreover, EVs isolated from media conditioned by primary human endometrial epithelial cells are efficiently internalized by human blastocysts and contain miRNAs directed toward genes involved in several processes related to embryo development [139]. Recent studies attempted to mimic in vivo conditions by sequentially culturing bovine embryos with EVs from oviductal and endometrial cells [140,141]. The addition of isthmus oviductal fluid EVs improved the development and cryotolerance of IVP blastocysts [141], whereas sequential culture with oviductal and endometrial EVs improved blastocyst cryotolerance by altering lipid content and metabolism, possibly in response to EVs’ miRNA contents [140].

While these studies deepen our understanding of embryo–maternal crosstalk during in vivo development, maternal EVs are obviously absent during in vitro embryo production, and the translation of such knowledge to in vitro embryo culture is still distant. Conversely, knowledge of the presence of embryo-derived EVs in vitro could be more readily applied to refine in vitro embryo culture in ART. Recent evidence indicates that embryo EVs may be internalized by the embryo itself, positively affecting its developmental competence. Pavani et al. (2018) [142] demonstrated that embryo EVs are taken up by zona-intact bovine embryos. Moreover, EVs extracted from media conditioned by high-density embryo group culture (25/50 μL) when added to individually cultured embryos increase blastocyst development to rates comparable to group culture, lower apoptotic cell ratios, ultimately demonstrating that EVs act as autocrine embryotropins during embryo culture. However, EVs from high-density group culture failed to increase the blastocyst hatching rates of individually cultured embryos to the same extent as reached under group culture. Generally, the developmental competence of singly cultured embryos is lower compared to group culture in terms of blastocyst cell number, hatching rates, and cryotolerance [143]. In addition, the hatching rates of IVD embryos are much higher than their IVP counterparts [45]. A recent study by the same group [144] isolated EVs from conditioned media of individually cultured embryos that either developed to the blastocyst stage or did not. Among the miRNAs differentially expressed in conditioned media from embryos that successfully developed or less to blastocyst, the authors selected bta-miR-378a-3p for further validation. Supplementation of the embryo culture medium with miR-378a-3p mimic significantly increased blastocyst cell numbers, reduced apoptotic cell numbers, improved hatching rates, and increased the expression of genes involved in embryo development and implantation. Knowledge of EVs derived from embryos may be translated into clinical practice to establish new non-invasive biomarkers for embryo selection in ART [5]. Further research on embryo EVs carrying miRNAs or other molecular cargos is warranted to enhance embryo development and quality and for selecting high-quality embryos [145].

In this scenario, confining single embryos into submicroliter volumes may be the key to success in concentrating autocrine embryotropins to levels similar to those present in vivo or achieved during high-density group embryo culture. However, attempts to reduce the volumes of drops under oil must address (1) nutrient depletion and waste metabolite accumulation; (2) the increase in surface-to-volume ratios that enhance unwanted bidirectional solute exchanges between oil and medium; (3) increased evaporation leading to osmolarity changes [2].

## 7. In Vitro Embryo Culture: Individual versus Group Culture

**Figure 2:**
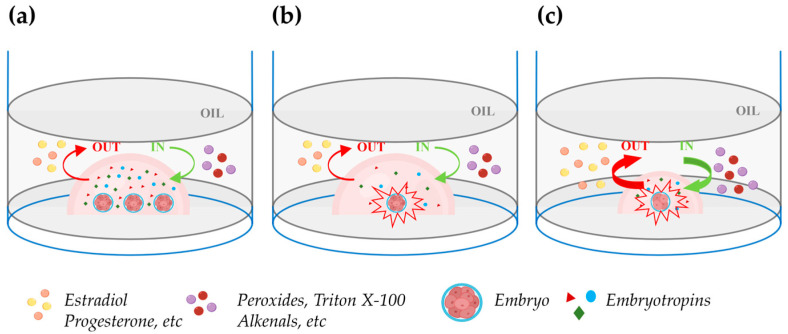
Impact of embryo density during in vitro culture in drops under oil. (**a**) Group embryo culture in a defined volume of culture medium improves embryo development due to an enhanced concentration of embryotropins (triangles, circles, and diamonds). The green and red curved arrows, respectively, indicate unwanted in (peroxides, Triton X-100, alkenals, etc.) and out (estradiol, progesterone, etc.) apolar solute exchanges between oil and medium. (**b**) Individual embryo culture within the same drop volume as in the figure (**a**): embryo development is compromised due to the reduced concentration of embryotropins. (**c**) Individual embryo culture in a reduced drop volume: embryotropins are more concentrated than in (**b**) and the increase in the drop surface exposed to oil relative to the drop volume exacerbates the unwanted in and out exchanges between the oil and medium, impairing embryo development. Figure created with BioRender.com (accessed on 11 April 2024).

It is well known that embryos benefit from being cultured together at high densities rather than individually in relatively large media volumes (Figure 2a,b). This phenomenon, termed the “group effect”, has been clearly recognized not only in mice, which are poly-ovulatory species, but also in domestic animals, which are largely mono-ovulatory. In multiple species, embryos cultured individually have been reported to exhibit slower cleavage divisions, reduced blastocyst rates and cell numbers, altered ICM-TE cell allocation, reduced hatching, increased apoptosis, altered gene expression, and reduced pregnancy rates [123,126,127,128,129,146] (Data are summarized in Table 3). The reduced blastocyst rates and cell numbers in embryos cultured individually might be explained by the low concentration of positive-effect embryo-secreted signaling molecules (Figure 2b). However, attempts to reduce the volume of drops under oil to achieve a high embryo density during individual culture generally yielded disappointing results.

Lane and Gardner (1992) [147] cultured single mouse embryos in drops of 5, 10, 20, or 320 μL and reported similar blastocyst rates but higher cell numbers in embryos individually cultured in 10 and 20 μL drops. Although the lower cell numbers in 320 μL of medium could result from low concentration of embryotropins, the same finding in 5 μL drops can be due to the increased surface exposed to oil relative to the drop volume which enhances unwanted damaging bidirectional solute exchanges between oil and medium (Figure 2c). In agreement with this hypothesis, individual culture in 5 μL in glass capillary tubes increased the blastocyst cell number to the same extent as in 10 or 20 μL drops. The beneficial effects of confinement in culture systems that avoid the harmful exchanges between oil and medium in reduced volumes microdrops was also elegantly shown by Thouas et al. (2003) [148] which cultured two mouse embryos in 1 µL glass capillaries achieving blastocyst cell numbers similar to those observed in high-density group culture with 20 zygotes in 10 μL drops under oil. Recently, Travaglione et al. (2024) showed that extreme confinement of individually cultured bovine embryos in ~70 nl microwells improved blastocyst rates compared to both conventional group culture (50 embryos/500 µL) and semi-confined group culture in microwell chambers (16 embryos/60 µL) [149]. Other studies showed that the spacing among embryos in high-density group cultures is critical. Stokes et al. (2005) [127] cultured groups of 16 IVD pig embryos in 20 μL drops at fixed distances and found maximal blastocyst and hatching blastocyst rates when the spacing was 81–160 μm. Lower rates were observed in drops with embryos in direct contact and no hatching blastocysts or blastocysts formed when the spacing was >480 and >640 μm, respectively. Similar findings were reported in the bovine model by Gopichandran and Leese (2006) [150]. Hoelker et al. (2009) [151] showed that group culture of 16 vs. 50 bovine zygotes in 500 μL of medium depresses the blastocyst rates and such effect is reverted when 16 zygotes are cultured in the same volume in a semi-confined well onto the well system.

**Table 3:** In vitro embryo culture: differences between individual versus group culture. WOW, Well of the Well.

Features	Embryo Density/Culture Conditions	Species	References
**Blastocyst Rate**	1/25 μL vs. 5 or 10/25 μL (49 vs. >80%)	Mouse	[123]
	1/25 μL vs. 1/50 μL (49 vs. 28%)	Mouse	[123]
	1/20 μL vs. 3–6/20 μL vs. 20/20 μL (0 vs. 6 vs. 23%)	Bovine	[126]
	16/20 μL different embryo–embryo distance (165 μm, ~20% vs. direct contact, ~8% vs. >540 μm, 0%)	Bovine	[150]
	16/500 μL vs. 50/500 μL vs. 16/500 μL WOW (21 vs. 32 vs. 31%)	Bovine	[151]
	50/500 μL vs. 16/60 μL MG vs. 1/68 nl MS (28.9 vs. 23.1 vs. 35.8%)	Bovine	[149]
	16/20 μL different embryo–embryo distance (81–160 μm, ~25% vs. >640 μm, 0%)	Porcine	[127]
	1/30 μL vs. 3–4/30 μL (45.2 vs. 55.8%)	Human	[129]
**Blastocyst Cell Numbers**	1/25 μL versus 5 or 10/25 μL (34 vs. >60)	Mouse	[123]
	1/20 μL vs. 20/20 μL (77 vs. 176)	Bovine	[126]
	16/20 μL different embryo–embryo distance (decreased cell numbers at 240 μm vs. 165 μm)	Bovine	[150]
	16/20 μL different embryo–embryo distance (1–160 μm, 49.9 vs. 401–480 μm, 32)	Porcine	[127]
	1/10 or 20 μL vs. 1/40 μL (75 vs. 60)	Mouse	[147]
	2/1 μL glass capillary vs. 20/10 μL droplet (92 vs. 75)	Mouse	[148]
**Blastocyst Hatching**	1/25 μL vs. 5 or 10/25 μL (10.7 vs. 52%)	Mouse	[123]
	1/2 μL vs. 10/20 μL (Day 4, 49 vs. 63%)	Mouse	[146]
	2/1 μL glass capillary vs. 20/10 μL droplet (48.3 vs. 3.3%)	Mouse	[148]
**Pregnancy Rate**	1/30 μL vs. 3–4/30 μL (30 vs. 60%)	Human	[129]
**Implantation Rate**	1/20 vs. 2/20 vs. 16/20 (27 vs. 32 vs. 38%)	Mouse	[147]

As mentioned, mineral oil can be harmful especially when embryos are cultured in small drops due to the increase in the surface exposed to oil relative to the drop volume (Figure 2c). In fact, early studies on oil embryotoxicity showed that mineral oil can absorb apolar solutes from the culture medium like estradiol, progesterone, and androstenedione [152]. A recent study showed that human blastocysts express both the enzymes required to synthesize estradiol and its receptors, and supplementation of embryo culture medium with 8nM estradiol improved formation of blastocyst cavity, blastocyst hatching, and the ICM/TE ratio but only when embryo culture was not performed under oil [153]. Mineral oil can also exert detrimental effects on embryo culture releasing a number of embryotoxic solutes into the culture medium like peroxides, alkenals, aldehydes, Triton X-100, and zinc [154,155,156,157,158]. In summary, high embryo density is the key of success to optimize embryo development but individual culture in drops of reduced volumes under oil is not the right way to increase the embryo density.

Recently, semiconfined individual embryo culture in microwell chambers based on the concept of the well onto the well originally developed by Vajta (2000) [159] has been largely adopted in human ART to follow each embryo individually through time-lapse morphokinetics. Culture of embryos in multiple microwells under one drop of shared medium should allow to concentrate putative, beneficial embryo-secreted factors in each microwell while the shared drop of medium should avoid the depletion of nutrients and the accumulation of waste metabolites. However, such devices preclude the possibility to non-invasively assess the competence of individual embryos through analysis of the spent media. Research in this field in the human and animal models clearly indicates the huge potential of non-invasive assessments on spent media to identify the developmental competence and the genetic status of individual embryos to transfer in a future ART setting [6,7,8].

## 8. Advanced Culture Systems to Mimic Fluid Mechanical Stimulation on In Vitro Embryo Culture

Recent studies have underscored the critical role of mechanical forces experienced by embryos within the oviduct and their implications for the optimization of in vitro embryo culture [160,161,162,163]. As aforementioned, in the dynamic environment of the oviduct, the embryo encounters a multitude of biomechanical cues and dynamic movements crucial for its transport and development. These include mechanical forces exerted by the oviduct wall, shear stress resulting from oviductal and subsequently intrauterine fluid pressure, as well as constriction from the peristaltic contractions of smooth muscle of the oviduct, and the metachronal ciliary wave of the oviductal cells resulting in a peristaltic-ciliary flow, which propel the embryo towards the uterus [92,104,164,165]. Understanding and replicating these biomechanical microenvironments in vitro have become areas of intense research focus, aiming to ultimately improve ART outcomes. Findings of the effects of advanced culture systems on embryo development in different species are summarized in Table 4.

Detailed investigations, particularly in the mouse model [186], revealed that to accurately mimic physio-mechanical conditions, sequential and distinct mechanical (i.e., shear stress, compressive strains, pressure) impulses may be necessary. However, early attempts to introduce a dynamic setup with continuous media perfusion at relatively high flow rates (0.1–0.5 µL/h) to culture embryos within microchannels (250 µm deep and 1 mm wide) yielded impaired embryo development, characterized by decreased proportions of morulae and blastocysts, and high abnormal eight-cell embryos compared to static microdevices [166]. Although the precise mechanism underlying this negative effect remains unclear, it could depend on physico-chemical factors including high shear stress on the embryo and/or the continual removal of growth-promoting autocrine factors [123,187]. Previous studies have proved that mouse embryos cultured in oil-suspended microdrops into rotating wall vessels (1.2 dynes/cm^2^ shear stress) exhibited an elevated susceptibility to shear stress beyond physiological levels in E2.5 embryos (compacted eight-cell/early morula stage) compared to E3.5 embryos (early blastocyst stage) and an up-regulation of stress-signaling pathway constituents. Furthermore, an increased lethality of embryos deprived of zona pellucida was observed after unphysiological shear-stress induction, demonstrating that excessive shear stress impairs embryo development compared with static embryo culture [168]. In this context, defining the magnitude of shear stress acting on human embryos during tubal transport poses a significant challenge, given the variability in reported velocities (average velocities 0.1 µm/s and maximum velocities around 8.6 µm/s) and uncertainties in extrapolating findings from animal models or womens’ oviductal tissue samples [188,189,190,191]. Establishing a safe range and duration of shear stress demands the development of precise, reliable, and controllable engineering systems. For instance, a low shear stress value <1.2 dyn/cm^2^ is often considered safe [188]. Incorporating a gentle surrounding fluid flow into embryo culture systems is highly complex [192,193]. In this direction, Heo et al. (2010) contributed to the integration of oviductal contractions and ciliary currents developing a micro-funnel with a physiologically-based pulsatile movement. Such a dynamic system improved mouse embryo development in terms of blastocysts hatching (Microfunnel-pulsatile 71 vs. 23% Microfunnel-control and 31% Microdrop-control), mean cell number (Microfunnel-pulsatile 109 + 5 vs. 60 + 3 Microfunnel-control, and 67 + 3 Microdrop-control) and pregnancy rates [169]. Other researchers emulated the in vivo compressive strain and duration of peristaltic constrictions through a micro-modulated syringe pump connected to a membrane-based microfluidic system highlighting the importance of the selection of the correct stimulus amplitude (150 Pa) and duration (2 s) in long-term bovine embryo culture [161]. Other studies have investigated the positive impact of physical stimulation, such as micro-vibration, to simulate the human Fallopian tube metachronal ciliary wave ranging from 5–20 Hz in vivo [194] on embryo development. Mizobe et al. (2010) observed a positive impact of micro-vibration on blastocyst rates when applied during in vitro maturation of pig oocytes [170]. In humans [171], mice [172], and bovines [173], increased blastocyst rates and reduced blastocysts mean cell numbers were observed with 40 Hz and 80 Hz frequencies, respectively. A detrimental effect of micro-vibration (20–42 Hz for 5 s/60 min) was reported on mouse embryos when applied during the early stage of embryo development in comparison with the control static group (47% vs. 56%, respectively) [174]. A recent investigation has suggested that micro-vibration (45 Hz for 5 s/60 min) induces an increased cryotolerance of bovine embryos along with improvements in epigenetic and transcriptional status in blastocysts [175]. A paired randomized controlled trial demonstrated that dynamic culture of human embryos with micro-vibration (42 Hz frequency for 5 min/60 min) did not improve formation of transferrable blastocyst (58.3% vs. 57.1%), aneuploidy rates (20.0% vs. 33.3%), or the blastocyst development rate (67.1% vs. 63.1%) vs. static groups, contrasting previous findings [176]. In striving to replicate mechanical stimuli akin to ciliary beating that move the embryo during transport within the female reproductive tract, a tilting embryo culture system (TECS) has been implemented. This approach demonstrated that estimated shear forces of ~0.7 dyn/mm^2^ improve blastocyst rates (mouse, TECS vs. control: embryo density 5/50 µL, 59 vs. 46%; embryo density 10/500 µL, 42% vs. 27%), cell numbers (TECS vs. control, mouse: 77 ± 4 vs. 66 ± 4; human: 43 ± 3 vs. 34 ± 3) in both mouse and human embryos [162,177]. An additional study explored the impact of the combined mechanical stimulations including a tilting culture system and straight microchannels (200 µm) with physical constrictions (150 or 160 µm) on bovine early embryo development, demonstrating an enhanced embryo cleavage (constricted vs. straight channel: 56.7 ± 13.7 vs. 23.9 ± 11.0%) with no differences in the blastocyst rates [178]. In the attempt to recapitulate the movements of embryos in the oviduct, a digitalized microfluidic device powered by electrowetting on a dielectric (EWOD), at a relatively low energy, was used to manipulate microculture droplets, demonstrating an enhancement of blastocyst hatching (dynamic vs. static: 50 vs. 21.2%) and the ability to produce normal live births in the mouse model [179]. However, concerns persist regarding the potential impact of the electric field on preimplantation embryos. While some studies suggest that certain levels of electrical stimulation (1.36 kV/cm for 40–60 ms) can enable fertilization in human oocytes [195], others indicate an increase in reactive oxygen species levels, for example in porcine embryos, exposed to electric pulses >1 kV/cm for 30 ms [180]. Another aspect to consider when trying to emulate in vitro the in vivo environment is the compression exerted by the oviductal wall on the embryo. The isthmus lumen diameter is relatively small, with certain sections being comparable to the diameter of a human embryo. Additionally, the uterine epithelium exhibits softer mechanical properties (10^2^–10^3^ Pa) compared to the conventional polystyrene petri dish (1 GPa) with a difference of nearly six orders of magnitude [181,196,197]. Advanced culture systems based on substitutive substrates have been developed in this direction by using static and/or dynamic microfluidic approaches as an alternative to conventional polystyrene petri dishes. Materials with different stiffnesses including polyacrylamide gels [183], agarose gels [182,184], alginate gels [185], PDMS [198] and collagen gels [181] were tested for their suitability for embryo culture, highlighting the positive impact of a low elastic modulus of the culture surface on the early preimplantation embryo developmental rate and allowing the in vitro post-implantation development. Accordingly, studies reported that the 3D type I collagen gels (1 kPa stiffness) culture system showed higher rates of blastocyst (64 ± 9.1 vs. 50 ± 18%), and hatching blastocyst stages (54 ± 25 vs. 21 ± 16%), as well as increased trophectodermal cell numbers (TE, 65 ± 13 vs. 49 ± 12 cells), compared to those cultured in a conventional polystyrene dish [181]. Furthermore, volume of media and oil may also influence forces exerted upon the embryo during various movement schemes, and factors such as friction or impact against platform edges may also be important variables. The use of microfluidic innovative systems has been proposed for customizing in vitro experiments for the manipulation of small volumes in the order of nanoliters to culture single embryos, recapitulating fluid volumes in the oviduct [199]. Microfluidic approaches that reduce volumes compared to the standard microdrop techniques have been demonstrated to have similar behavior in terms of blastocyst attachment (33.22 ± 5.6 vs. 45.64 ± 8.4%) [199] or an enhanced embryo development [167]. Future studies introducing fluid flow to such systems in which autocrine and paracrine factors are concentrated in reduced volumes could lead to higher pregnancy and implantation rates in ART. All these considerations lead us to understand that an optimal embryo culture system should incorporate the capacity to mimic, at a microscale, wide-ranging consistencies, mechanical forces, and manipulation of minute volumes in the nanoliter range, all essential aspects for accurately recapitulating embryo development in vitro and gaining widespread embryo culture implementation [163,167].

## 9. Conclusions

Current embryo culture systems in ART fail to accurately replicate the biophysical factors and signaling cross-talk that embryos experience in vivo. The consequences of such discrepancies on the developmental competence of in vitro-produced embryos compared to their in vivo counterparts are challenging to discern in human ART. However, research in animal models, particularly in bovine species, more clearly demonstrates the lower developmental competence and higher genomic instability of IVP embryos compared to IVD embryos.

Three main factors of the in vivo environment are currently not replicated in in vitro embryo culture systems: (1) the biophysical conditions experienced by embryos in the maternal tract, such as oviductal fluid flow, metachronal ciliary waves, peristalsis, and the low elastic modulus of biological surfaces in contact with the embryo; (2) oviductal and endometrial soluble embryotropins or other molecular cargos enclosed in extracellular vesicles that have positive effects on embryo development; (3) confinement in sub-microliter volumes that enhances the concentration of embryo-secreted factors beneficial to the embryo.

In animal models, in vitro embryo development is generally improved under high embryo density conditions. However, in human ART, the relatively low number of embryos and the need to monitor the development of individual embryos preclude the possibility of in vitro culture at high embryo densities. Semi-confined culture in well-of-the-well devices has been widely adopted in human ART to allow the collection of morphokinetic data of individual embryos. Nevertheless, such culture systems do not permit the collection of individual embryo spent culture media for non-invasive analysis of embryo developmental competence and euploidy.

Although several research studies indicate that dynamic culture systems can improve embryo developmental competence, we are still far from faithfully recapitulating the high and poorly understood complexity of biophysical factors acting during in vivo embryo development. Nevertheless, the increasing interest of research groups in this field holds promise for establishing a more physiological, confined, and dynamic individual embryo culture system allowing the collection and analysis of non-invasive markers of embryo developmental competence.

## Figures and Tables

**Table 1:** Mouse animal model: differences between IVP and IVD embryos. ICM, inner cell mass; TE, trophectoderm; ROS, reactive oxygen species; GSH, reduced glutathione; Mmp-9, matrix metalloproteinase-9.

Features	IVP versus IVD Embryos	References
**Blastocyst cell numbers**	Lower TE, lesser reduction of ICM	[24]
	Lower cell numbers	[25]
	Altered TE and/or ICM cell numbers	[26]
**Blastocyst hatching**	Lower hatching rate	[27]
**Apoptosis**	Increased TUNEL-positive cells	[28]
**Cryotolerance**	Lower survival of 2-cell embryos after warming	[29]
**Energy metabolism**	Decreased mitochondrial activity and increased glycolytic function	[25]
	Mitochondrial dysfunctions: dysregulation of 806 mitochondria-related genes validated through cytological/molecular analysis	[28]
**Oxidative stress**	Increased ROS; decreased GSH	[25,28]
	Increased oxidative damage of DNA, lipids, and proteins	[25]
**Gene expression**	Lower (~10.7-fold) expression of Mmp-9 required for successful implantation and trophoblast invasion	[27]
	Differential expression of 1000 genes involved in proliferation, apoptosis, and morphogenetic pathways	[29]
	Differential expression of 1912 genes (29 > 4-fold) involved in proliferation, apoptosis, and morphogenetic pathways	[24]
	Differential expression of genes involved in metabolism, proliferation, apoptosis, and morphogenetic pathways	[26]
	Differential expression of genes involved in cytoskeletal organization	[30]

**Table 2:** Bovine animal model: differences between IVP and IVD embryos. ICM, inner cell mass; TE, trophectoderm.

Features	IVP vs. IVD Embryos	References
**Pregnancy Rate**	~ 40.1 vs. 64.1%	[33]
**Blastocyst Rate**	Lower blastocyst yield and quality	[45]
**Hatching Rates**	Lower hatching rate	[45]
**Blastocyst Cell Number**	Altered ICM/TE allocation	[46]
**Apoptosis**	Higher apoptosis rate	[35]
**Cryotolerance**	Reduced blastocyst cavity re-expansion after vitrification	[35]
	Increased cryodamage	[43]
	Lower survival after warming	[45]
**Lipid Content**	Higher lipid accumulation and down-regulation of lipid metabolism genes	[35,39,48]
**Developmental Competence**	Lower developmental competence	[39,52,53,54,55,56,57,58]
**Aneuploidy**	Higher chromosome aberrations	[56,57]
**Ultrastructure**	Differences in morphology and cell structure	[42,43,44,45]
	Reduced intercellular connectivity	[47]
**Energy Metabolism**	Differences in energy metabolism profile	[51]
**Transcriptomic**	Reduced oxidative stress response	[39]
	Down-regulation of lipid metabolism genes	[39]
	Differential expression of 793 genes, differential alternative splicing of 873 genes	[52]
	Lower abundance of peroxiredoxin 1, heat shock protein 70.1, growth and differentiation factor 9, and maternal antigen	[53]
**Epigenetics**	Hypomethylated genomic loci in blastocysts	[58]
	Increased Large Offspring Syndrome	[54]
**Proteomic**	Lower abundance of proteins related to carbohydrate metabolism and cytoplasmic cellular components Higher abundance of translation-related proteins after the morula stage	[55]

**Table 4:** Advanced culture systems designed to replicate the biomechanical microenvironment of the maternal reproductive tract. ROS, reactive oxygen species; TE, trophectoderm.

Advanced Culture Systems	Microenvironmental Feature Mimicked	Key Findings	References
**Microchannel System**	Continuous media perfusion flow rates 0.1–0.5 µL/h	Impaired mouse embryo development	[166]
	No media perfusion	Improved mouse embryo cleavage and blastocyst rates	[167]
**Rotating Wall Vessels**	Shear stress (1.2 dynes/cm^2^)	Impaired mouse embryo development Up-regulation of stress signaling pathways	[168]
**Micro-funnel with Pulsatile Movement**	Peristaltic contractions and metachronal ciliary wave	Improved mouse blastocyst hatching, mean cell numbers, and pregnancy rates	[169]
**Micro-modulated Syringe Pump**	Compressive strain and duration of peristaltic contractions	Improved 8-cell stage bovine embryo development	[161]
**Micro-vibration Systems**	Metachronal ciliary wave (5 s/30–60 min or 10 s/60 min)	Vibration during in vitro maturation improves porcine blastocyst rates	[170]
	Metachronal ciliary wave (44 Hz, 5 s/60 min duration)	Improved day 3 embryo quality, blastocyst, and pregnancy rates in human	[171]
	Metachronal ciliary wave (42 Hz, 5 s/60 min duration)	Improved 8-cell embryo rate, hatched blastocyst rate, and blastocyst cell numbers in mouse Improved human blastocyst rates, implantation, and pregnancy rates	[172]
	Metachronal ciliary wave (5 s/60 min at 20, 40, or 80 Hz)	Increased bovine blastocyst rates at 40 Hz Reduced blastocysts cell number at 80 Hz	[173]
	Metachronal ciliary wave (20–42 Hz for 5 s/60 min)	Reduced mouse blastocyst rates	[174]
	Metachronal ciliary wave (45 Hz for 5 s/60 min)	Improved bovine blastocyst cryotolerance, epigenetic, and transcriptional status	[175]
	Metachronal ciliary wave (42 Hz for 5 min/60 min)	No improvements of human blastocyst and aneuploidy rates	[176]
**Tilting Embryo Culture System (TECS)**	Metachronal ciliary wave	Improved blastocyst rates and cell numbers in both mouse and human embryos	[162,177]
**TECS/Microchannels**	Metachronal ciliary wave associated with straight microchannels (200 μm) with physical constrictions (150 or 160 μm)	Improved bovine embryo cleavage	[178]
**Microfluidic Devices with Electrowetting (EWOD)**	Oviductal embryo transport (electrical pulses—60~68.5 VRMS at 500 Hz)	Improved mouse blastocyst hatching Healthy live births	[179]
	Oviductal embryo transport (electrical pulses > 1 kV/cm for 30 ms)	No improvements of porcine cleavage and blastocyst rates Increased ROS levels	[180]
**Microfluidic Systems (Static or dynamic)**	Softness of oviductal wall	Improved mouse blastocyst and hatching rates, and TE cell numbers	[181]
		Improves culture of bovine and ovine zona-free nuclear-transfer embryos	[182]
	Softness of uterine wall	Enables early post-implantation mouse embryo development	[183]
		Enables post-hatching culture of bovine embryos	[184,185]

## Data Availability

Data are freely available from the authors.
